# Prevalence of *PNPLA1* Gene Mutation in 48 Breeding Golden Retriever Dogs

**DOI:** 10.3390/vetsci5020048

**Published:** 2018-05-08

**Authors:** Lisa Graziano, Mauro Vasconi, Luisa Cornegliani

**Affiliations:** 1 Clinica Veterinaria Meda, via Libertà 76, 20825 Milan, Italy; 2 Department of Health, Animal Science and Food Safety, University of Milan, via Trentacoste 2, 20134 Milan, Italy; mauro.vasconi@unimi.it; 3 Clinica Veterinaria San Siro, via Lampugnano 99, 20151 Milan, Italy; lcornegliani@libero.it

**Keywords:** breeding, genetic disorder, Golden Retriever, *PNPLA1*, ichthyosis

## Abstract

A non-epidermolytic ichthyosis has been identified in Golden Retrievers due to a variant in the *PNPLA1* gene, and a genetic test is available to detect wild-type, heterozygous and homozygous dogs. The aims of this study were to investigate the prevalence of the *PNPLA1* gene variant in Golden Retrievers used for breeding and to provide more information to breeders in order to restrict the spread of this disease. Clinical examination and assessment of the *PNPLA1* genotype using PCR testing of oral swabs were performed in 48 breeding Golden Retrievers. Wild-type, heterozygous or homozygous variants of the *PNPLA1* gene were demonstrated in 10 (21%), 23 (48%), and 15 (31%) of the 48 dogs, respectively. In only 3 of the 48 dogs were clinical signs suggestive of ichthyosis identified. Data collected agreed with data reported in the literature. The high prevalence of homozygous and heterozygous variants makes the exclusion of mutated dogs from breeding impractical. Furthermore, the reliability of the *PNPLA1* mutation in prediction of clinical signs of ichthyosis is unclear. Additional studies are needed to investigate if *PNPLA1* is the only gene involved or if other genes and environmental factors have a role in the development of ichthyosis in Golden Retrievers.

## 1. Introduction

Ichthyosis is a genodermatosis that affects humans, dogs and, rarely, cats. In veterinary medicine, ichthyosis is classified into epidermolytic and non-epidermolytic subtypes [1].

Epidermolytic ichthyosis has been identified in Norfolk terriers due to a defect in keratin 10, because of a splice site mutation [2]. It is characterized by diffuse ballooning of keratinocytes above the basal layer [1]. Non-epidermolytic ichthyosis has been found in Golden Retrievers, Jack Russell terriers, American bulldogs, German Shepherd, and other breeds [3,4,5,6] and is characterized by the absence of vacuolization or lysis of keratinocytes [2,7].

In Golden Retrievers, non-epidermolytic ichthyosis is associated with variants in the *PNPLA1* gene [8]. The mode of inheritance is autosomal recessive [3,4,7,8].

A commercial gene test has been released to identify the *PNPLA1* gene variant [9].

The aims of this study were to: (i) investigate the prevalence of the *PNPLA1* gene variant in Golden Retriever dogs selected for breeding in Italy; and (ii) provide more information to breeders, in order to exclude affected dogs from breeding programs and restrict the spread of this disease. 

## 2. Materials and Methods

This study was conducted in accordance with the principles of Good Clinical Practice (G.U. No. 289, 10-12-1996, 47-53), and written informed consent was obtained from all breeders involved.

### 2.1. Inclusion and Exclusion Criteria

Italian breeders were contacted, and a total of 48 breeding Golden Retrievers between 1 and 13 years of age were enrolled in the trial. First-degree relatives were excluded. At the same time, dogs with severe clinical signs of ichthyosis were not part of this cohort as they generally are not used as potential breeding animals.

### 2.2. Clinical Records

Dermatological examination of all of the dogs was performed by dermatologists; clinical signs suggestive of ichthyosis, that is, nonpruritic scaling (dandruff) and hyperpigmentation affecting ventral regions and limbs with symmetrical distribution, were recorded. Breeders were asked about the presence of previous skin disorders in these dogs, especially in puppyhood.

### 2.3. Sampling and Shipping

Dogs were fasted for 1 hour before cells collection, and water consumption was not permitted 30 min before the sampling. Forty-eight oral mucosal swabs were collected with a cytobrush, with a firm rotation of the brush between cheek and gingiva for 20 s. After the collection, the brush was immersed in a test tube filled with ethanol. Each sample was identified by a number and a certificate containing breeder and dog data. Refrigerated samples were then shipped to the Antagene laboratory. 

### 2.4. DNA Analysis

DNA extraction and amplification was performed by PCR technique by Antagene laboratory that produced the commercial test. A gene test for the *PNPLA1* variant was performed as previously described [9]. According to the laboratory, dogs with the wild-type gene are unaffected and there is no transmission of the variant to their lineage. Heterozygous dogs are unaffected and could transmit the *PNPLA1* variant to 50% of their lineage. Homozygous dogs are affected with clinical signs and will transmit the *PNPLA1* variant to 100% of their lineage [9].

### 2.5. Statistical Analysis

Prevalence rates were compared by means of Pearson’s chi-squared test, using the Yates correction for continuity. Results are presented as odds ratios (Ors) for the presence of the *PNPLA1* variant. Results of our study were compared with two analog studies [10,11] using the same test used before. For the comparison of our results with [11], only female *PNPLA1* prevalence was used. Significance was set at *p* < 0.05, and all analyses were performed with statistical software SPSS 22 (IBM, Armonk, NY, USA).

## 3. Results

Forty-eight breeding Golden Retriever dogs were enrolled in this study. Among them, 20 dogs were male and 28 female. Results of the gene test for the *PNPLA1* variant are presented in Table 1. The prevalence of carriers (or heterozygotes) was 48% followed by those homozygous for the variant at 31%, and wild-type at 21%. Statistical analysis showed a significant difference between males and females that presented the homozygous variant of the *PNPLA1* gene in the studied population. This condition was detected mainly in females. Thirteen of 28 Golden Retriever bitches (46%) presented the homozygous variant while only 2 of 20 males presented the same gene variant condition. In only 3 of 48 dogs were clinical signs suggestive of ichthyosis identified.

## 4. Discussion

Non-epidermolytic ichthyosis has been recognized in Golden Retriever dogs since 2000. It is characterized clinically by small to large, whitish to brownish scales. Histopathological features include moderate to severe, laminated, or compact orthokeratotic epidermal hyperkeratosis with absent to minimal epidermal hyperplasia, and no dermal inflammation [4,7]. Non-epidermolytic ichthyosis in Golden Retrievers is characterized by juvenile onset, usually prior to 1 year of age, of moderate to severe diffuse rarely pruritic exfoliation and hyperpigmentation (sandpaper-like appearance). The distribution of lesions is symmetrical and mainly affects axilla, thoracic, flanks and inguinal regions; the nasal planum, footpads, and ear canals are usually spared [2,4].

The most common differential diagnosis includes parasitic disorders, sebaceous adenitis, hypothyrodism, and cutaneous lymphoma [5].

The diagnosis is routinely based on clinical signs, ruling out other causes of seborrhea, and histopathological findings [3].

Ichthyosis in Golden Retrievers was suggested to be an autosomal recessive disorder caused by a variant in the *PNPLA1* gene [3,4,6,7]. This gene belongs to the patatin-like phospholipase [PNPLA] family that includes 9 members (PNPLA 1–9) with key roles in lipid metabolism and signaling [12]. In Golden Retrievers, the *PNPLA1* gene variant causes truncation within the highly conserved C-terminal region. The indel mutation induces a premature stop-codon with loss of 74 amino acids and is assumed to lead to a non-functional protein causing alterations in the keratinization process [8]. A genetic test was developed to identify this mutation through buccal swabs or whole blood [9].

In our study we investigated the prevalence of the *PNPLA1* gene variant in Golden Retrievers selected for breeding in Italy. Among our dogs, the wild-type, heterozygous and homozygous variants of the *PNPLA1* gene were observed in in 10 (21%), 23 (48%), and 15 (31%) of the 48 dogs, respectively.

Statistical analysis showed a significant difference between sex distribution of the homozygous variant of the *PNPLA1* gene in the studied population, with a greater prevalence in bitches (*p* < 0.05). This result could be related to the largest number of females used for breeding and a more accurate selection of sires, excluding over time the ones clinically affected.

Data collected were not statistically different (*p* > 0.05) from data reported in the literature [10,11].

Owczarek-Lipska et al, 2011 studied the frequency of gene defects in selected European Retriever populations in Switzerland. Among the 179 dogs subjected to genetic investigation, they found 35 (20%) wild-type dogs, 87 (49%) heterozygous, and 57 (32%) homozygous dogs for the *PNPLA1* variant [10]. 

In Germany, a wild-type, heterozygous, or homozygous variant of the *PNPLA1* gene was demonstrated in 19%, 53%, and 28% of Golden Retriever puppies, respectively. In the same study a wild-type, heterozygous, or homozygous mutant of the *PNPLA1* gene was demonstrated in 18%, 47%, and 35% of adult dogs, respectively [11].

In our study 3 of 48 dogs (6%) had clinical signs suggestive of ichthyosis while 45 of 48 (94%) had no clinical signs at the time of examination or a preceding history of skin lesions. All of the affected dogs were identified as homozygous. 

These results are in agreement with those published in a previous study that showed only a portion of homozygous dogs had scales with or without hyperpigmentation (69%) [13].

The high percentage of our homozyous healtly dogs could be overestimated because severely affected dogs were excluded during the inclusion, minimal clinical signs may have been overlooked during the examination, and clinical signs may disappear in older dogs. 

In our experience, ichthyosis is rarely considered a disease and mild to moderate scales or hyperpigmentation are not perceived as symptoms by breeders, so another reason could be found in wrong information obtained by breeders regarding skin lesions in puppyhood. Recently, a study demonstrated homozygous puppies could exhibit clinical signs suggestive of ichthyosis prior to 1 year of age and become subclinical in adulthood suggesting that the *PNPLA1* genotype is more closely correlated with the clinical phenotype at a young age than in adulthood [11].

Our data are not in complete agreement with Grall et al., 2012 who found all homozygous *PNPLA1* mutant dogs to be affected [8].

In conclusion, our study shows that the prevalence of the *PNPLA1* gene variant in Italian breeding programs is high and that only 21% of sampled dogs represent the wild-type. In Italy the *PNPLA1* variant gene test is not commonly used by breeders to choose dogs for reproduction because of the cost of the test and lower interest compared with other breeding disorders. This means heterozygous and homozygous dogs are not recognized and some with mild clinical signs are used for breeding. This contributes to the spread of the variant. 

However, the exclusion of all homozygous and heterozygous Golden Retrievers from breeding may be impractical because of the high prevalence of this variant, as suggested in previous studies [11,13]. 

Another significant issue is the unreliability of the *PNPLA1* mutation in prediction of clinical manifestation of ichthyosis. 

At the present time the exclusion of Golden Retrievers with clinical signs from reproduction is highly recommended. It could be wise to use at least one clear animal in each mating. This restriction would bring down the percentage of homozygous mutant dogs and thus the prevalence of the disease. Particularly affected sires should always be excluded due to the possibility of major number of annual matings. 

Further studies are needed to better investigate whether *PNPLA1* is the only gene involved or if other genes or environmental factors have a crucial role in the evolution and development of ichthyosis in Golden Retrievers dogs, or if the *PNPLA1* variation merely represents a predisposition rather than a direct cause of the disease [3,13].

## Figures and Tables

**Table 1:** Genotype of 48 Golden Retriever for the *PNPLA1* gene mutation.

	n	Wild Type	Heterozygous	Homozygous
Male	20 (42%)	6 (30%)	12 (60%)	2 (10%)
Female	28 (54%)	4 (14%)	11 (39%)	13 (46%)
Total	48	10 (21%)	23 (48%)	15 (31%)

## References

[1] Gross T.L., Ihrke P.J., Walder E.J., Affolter V.K., Gross T.L., Ihrke P.J., Walder E.J., Affolter V.K. (2005). Diseases with abnormal cornification. Skin Diseases of the Dog and the Cat—Clinical and Histopathological Diagnosis.

[2] Credille K.M., Barnhart K.F., Minor J.S., Dunstan R.W. (2005). Mild recessive epidermolysis hyperkeratosis associated with a keratin 10 donor splice site variant in a family of Norfolk terrier dogs. Br. J. Dermatol..

[3] Mauldin E.A., Credille K.M., Dunstan R.W., Casal M.L. (2008). The clinical and morphologic features of nonepidermolytic ichthyosis in the Golden Retriever. Vet. Pathol..

[4] Cadiergues M.-C., Patel A., Shearer D.H., Fermor R., Miah S., Hendricks A. (2008). Cornification defect in the Golden Retriever: Clinical, histopathological, ultrastructural and genetic characterization. Vet. Dermatol.

[5] Mauldin E.A. (2013). Canine ichthyosis and related disorders of cornification in small animals. Vet. Clin. N. Am. Small Anim. Pract..

[6] Bauer A., Waluk D.P., Galichet A., Timm K., Jagannathan V., Sayar B.S., Wiener D.J., Dietschi E., Müller E.J., Roosje P. (2017). A de novo variant in the ASPRV1 gene in a dog with ichthyosis. PLoS Genet..

[7] Guaguere E., Bensignor E., Küry S., Degorce-Rubiales F., Muller A., Herbin L., Fontaine J., André C. (2009). Clinical, histopathological and genetic data of ichthyosis in Golden Retriever: A prospective study. J. Small Anim. Pract..

[8] Grall A., Guaguere E., Planchais S., Grond S., Bourrat E., Hausser I., Hitte C., Le Gallo M., Derbois C., Kim G.J. (2012). *PNPLA1* variants cause autosomal recessive congenital ichthyosis in Golden Retriever dogs and human. Nat. Genet..

[9] Antagene Laboratory Website Ichthyosis of Golden Retriever Breed. www.antagene.com/en/ict-ichthyosisGolden-Retriever.

[10] Owczarek-Lipska M., Thomas A., Andrè C., Hölzer S., Leeb T. (2011). Häufigkeit von Gendefekten in ausgewählten europäischen Retriever Populationen. Schweiz. Arch. Tierheilkd..

[11] Roething A., Schildt K.J., Welle M.M., Wildermuth B.E., Neiger R., Thom N. (2015). Is “milk crust” a transient form of Golden Retriever ichthyosis?. Vet. Dermatol..

[12] Kienesberger P.C., Oberer M., Lass A., Zechner R. (2009). Mammalian patatin domain containing proteins: A family with diverse lipolytic activities involved in multiple biological functions. J. Lipid Res..

[13] Jensen C.N. (2014). Ichthyosis in the Golden Retriever—Variation of Clinical Manifestations and Breeding Aspects in Golden Retrievers that Are Homozygous for the PNPLA1 Variant. Master’s Thesis.

